# Increased Minimum Mortality Temperature in France: Data Suggest Humans Are Adapting to Climate Change

**DOI:** 10.1289/ehp.123-A184

**Published:** 2015-07-01

**Authors:** Julia R. Barrett

**Affiliations:** Julia R. Barrett, MS, ELS, a Madison, WI–based science writer and editor, is a member of the National Association of Science Writers and the Board of Editors in the Life Sciences.

Outdoor temperatures can influence population mortality rates, with the mortality–temperature relationship typically depicted as a U- or J-shaped curve.^1^ The lowest point of this curve defines the minimum mortality temperature (MMT)—that is, the temperature with the lowest mortality rate—while the raised ends represent increased deaths at both lower and higher temperatures. A new study in this issue of *EHP* that’s based on 42 years’ worth of climatic and mortality data shows that the MMT in France has increased over time, suggesting some measure of adaptation to warming during that period.^1^

Climate change models predict higher average temperatures and more frequent and intense heat waves in the coming decades.^2^^,^^3^ Hot weather contributes to potentially deadly heat exhaustion and heat stroke, and can exacerbate preexisting medical conditions. This is particularly true for populations that are vulnerable due to age, socioeconomic factors, limited social support, geographic isolation (for instance, rural areas where air conditioning may be less common), and poor access to health care.^2^^,^^3^^,^^4^^,^^5^ Higher rates of heat-related deaths tend to occur with heat waves earlier in the summer, before people have acclimatized to the season’s hotter temperatures.^6^ Improvements in social, environmental, behavioral, and health care factors can reduce a population’s vulnerability,^7^ and acclimatization to warmer temperatures may also reduce heat-related impacts on human health.^2^^,^^5^

**Figure:**
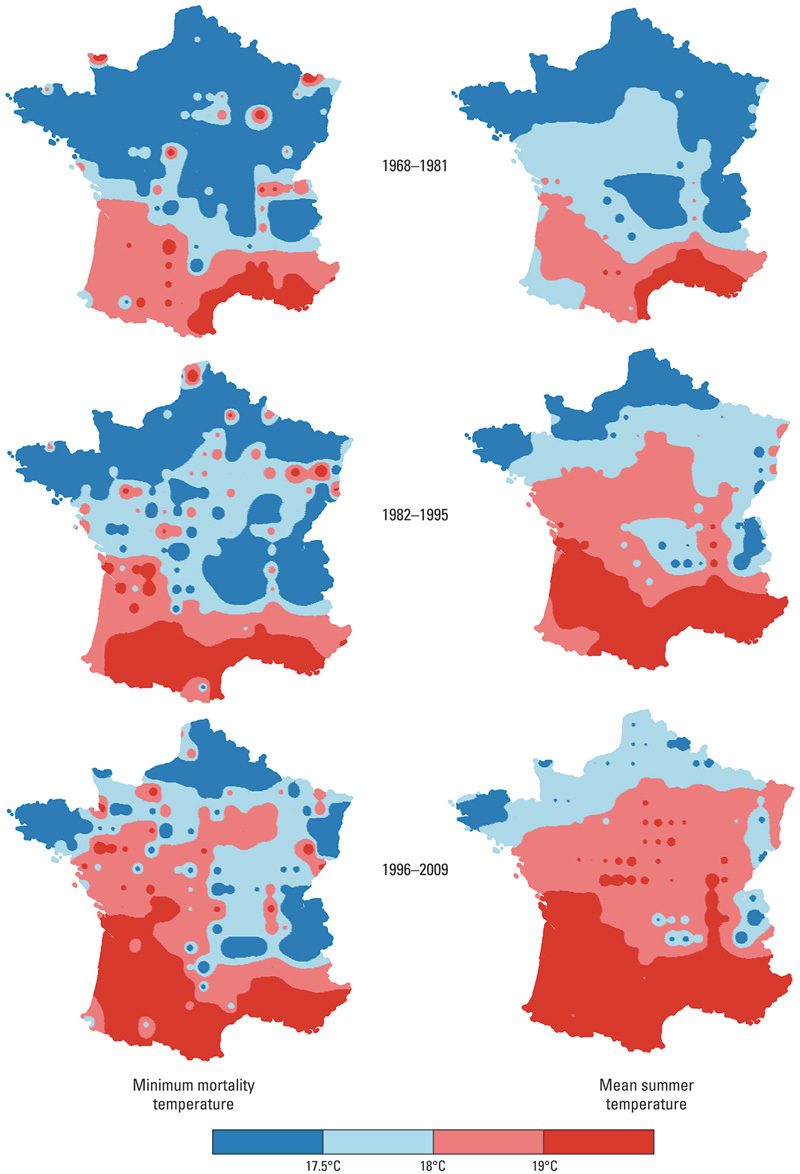
As mean summer temperatures in France increased over time (right), so did the temperatures associated with the lowest mortality rates (left). Source: Todd and Valleron (2015)^1^

The current study demonstrates how statistical modeling paired with the increasing availability of enormous data sets might predict the extent to which humans can adapt to climate change. Unlike previous work exploring the temperature–mortality relationship, this study included small towns and rural settings as well as urban centers. The researchers used high-resolution climate data to superimpose a grid of 30×30-km squares over continental France. The resultant 295 squares encompassed 36,000 *communes*, the smallest administrative unit for which sociodemographic data were available.

The researchers obtained death certificates for all people aged 65 and older who died of natural causes within continental France during 1968–2009, a total of 16,487,668 deaths. “Temperature influences many causes of deaths,” explains coauthor Alain-Jacques Valleron, emeritus professor at Université Pierre et Marie Curie. “We would have lost much of what we wanted to study—the impact of temperature on mortality—if we had only focused on deaths that were registered as directly related to temperature.”

Analyses of the temperature–mortality relationship focused on grid squares with more than 22,500 deaths during the entire 42-year period (228 squares). The researchers also divided the study period into three 14-year periods (1968–1981, 1982–1995, and 1996–2009), focusing on squares with more than 7,500 deaths during each period (224 squares).

Nearly all squares had U- or J-shaped temperature–mortality curves. The MMT was found to shift upward over time, from 17.5°C (63.5°F) in the first period, to 17.8°C (64.0°F) in the second period, to 18.2°C (64.8°F) in the third period. Mean summer and winter temperatures also increased over time, by 1.6°C (2.9°F) for summer and 0.8°C (1.4°F) for winter. Subsequent sensitivity analyses corroborated these results. The authors concluded that adaptation to higher temperature had occurred during the 42-year period of the study.^1^

“I was interested in their conclusions about the MMT shift in time,” says Matthew Heaton, an assistant professor of statistics at Brigham Young University who was not involved in the study. “I think it’s a nice conclusion, but I’d like to see more to justify their conclusions—for example, confidence intervals or hypothesis testing.”

Heaton also praises the length of time covered by the study, “the longest that I’ve seen,” and the fact that it covers rural areas. He points out that some of the analyses focused on locations that had more than 15,000 deaths, which cuts out the least populated areas. However, those analyses did not change the overall conclusions.

The researchers are planning to expand on their findings, says Valleron, particularly with regard to the causes of mortality and in light of temperatures plateauing in some places within France. As the authors note, their study considered only one aspect of climate change that could affect health—temperature. Temperature-related impacts may coexist with other factors affecting mortality rates.^2^ However, these factors can also be examined using models similar to those in the current study. Says Valleron, “The approach can be extended to many environmental databases to try to find environmental causes of disease.”
